# Trans-species polymorphism in humans and the great apes is generally maintained by balancing selection that modulates the host immune response

**DOI:** 10.1186/s40246-015-0043-1

**Published:** 2015-09-04

**Authors:** Luisa Azevedo, Catarina Serrano, Antonio Amorim, David N. Cooper

**Affiliations:** Instituto de Investigação e Inovação em Saúde, Universidade do Porto, Porto, Portugal; IPATIMUP-Institute of Molecular Pathology and Immunology, University of Porto, Rua Dr. Roberto Frias s/n, 4200-465, Porto, Portugal; Department of Biology, Faculty of Sciences, University of Porto, Rua do Campo Alegre, s/n, 4169-007, Porto, Portugal; Institute of Medical Genetics, School of Medicine, Cardiff University, Heath Park, Cardiff, CF14 4XN, UK

## Abstract

Known examples of ancient identical-by-descent genetic variants being shared between evolutionarily related species, known as trans-species polymorphisms (TSPs), result from counterbalancing selective forces acting on target genes to confer resistance against infectious agents. To date, putative TSPs between humans and other primate species have been identified for the highly polymorphic major histocompatibility complex (MHC), the histo-blood ABO group, two antiviral genes (*ZC3HAV1* and *TRIM5*), an autoimmunity-related gene *LAD1* and several non-coding genomic segments with a putative regulatory role. Although the number of well-characterized TSPs under long-term balancing selection is still very small, these examples are connected by a common thread, namely that they involve genes with key roles in the immune system and, in heterozygosity, appear to confer genetic resistance to pathogens. Here, we review known cases of shared polymorphism that appear to be under long-term balancing selection in humans and the great apes. Although the specific selective agent(s) responsible are still unknown, these TSPs may nevertheless be seen as constituting important adaptive events that have occurred during the evolution of the primate immune system.

## Introduction

Trans-species polymorphisms (TSPs) are ancient genetic variants whose origin predates speciation events, resulting in shared alleles between evolutionarily related species [1]. Shared polymorphisms are only considered to be TSPs sensu stricto when there is convincing evidence to show that they are identical-by-descent rather than recurrent mutations occurring independently in different lineages (i.e. identical-by-state); the latter often involve the CpG dinucleotide [2, 3] whose hypermutability is directly attributable to its role as the major site of cytosine methylation, with the attendant risk of spontaneous deamination of 5-methylcytosine to yield thymine. It follows that, even in a genomic region which manifests signals of balancing selection, a specific shared polymorphism that is located within a CpG dinucleotide is, by its very nature, more likely to be identical-by-state due to recurrent mutation in distinct lineages rather than it is to be identical-by-descent. In such cases, any of the strong signals of balancing selection observed may emanate from functionally relevant balanced polymorphisms that are closely linked to the CpG site in question but which are not themselves shared across lineages.

**Fig. 1:**
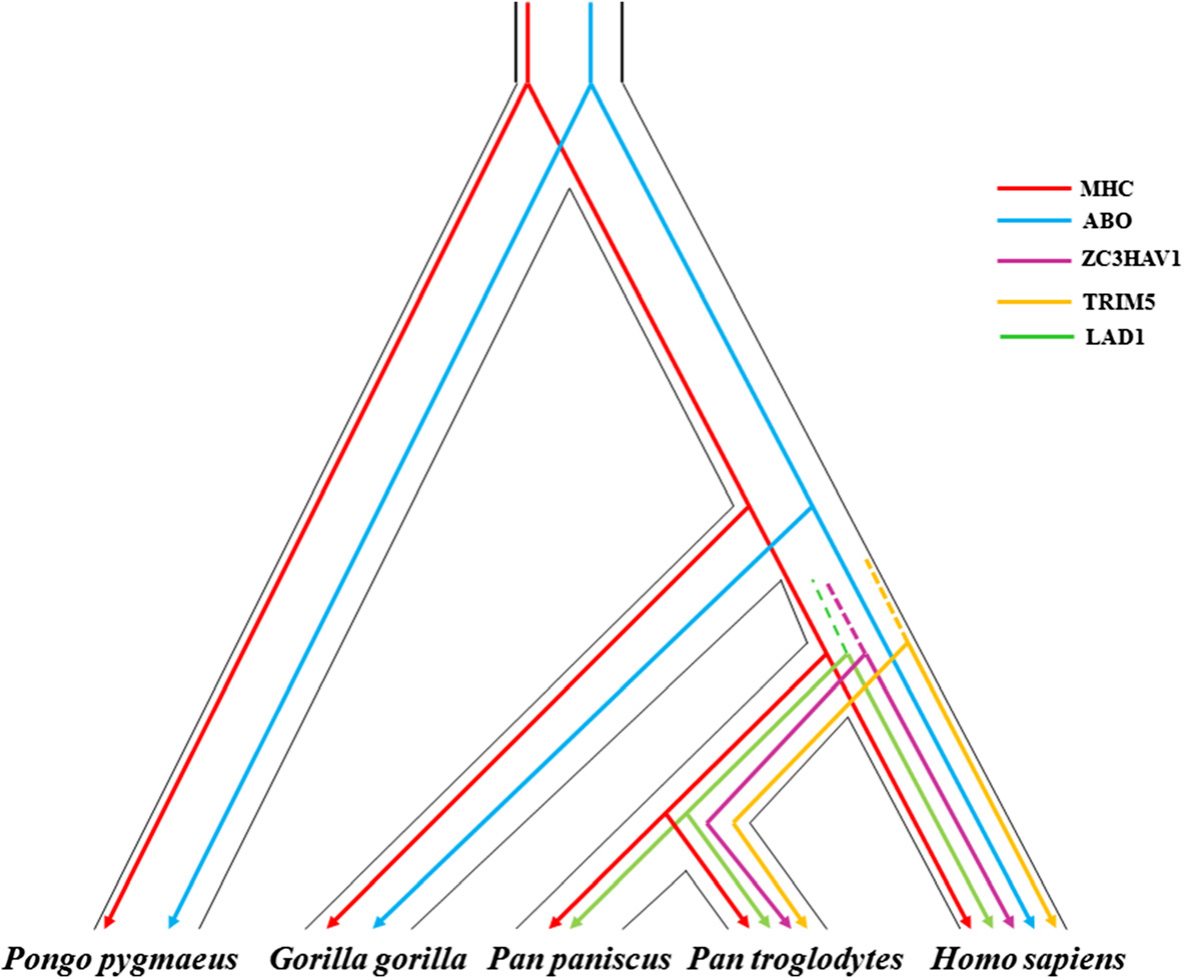
Known examples of trans-species polymorphisms maintained by long-term balancing selection in humans and the great apes

The long-term (i.e. post-speciation) preservation of bona fide identical-by-descendent TSPs is inherently unlikely under a purely neutralist model of evolution [4, 5], and hence the action of selection must invariably be assumed. In passing, it should be noted that positive selection is also implicit in the case of independently occurring identical-by-state polymorphisms in different lineages. Here, we review the small number of relatively well-characterized examples of TSPs that are shared between the genomes of great apes (human, common chimpanzee, bonobo, gorilla and orangutan), focusing specifically on those for which there is a body of evidence for long-standing balancing selection (Fig. 1).

### TSPs maintained by balancing selection: the MHC and ABO loci

Evidence for the common ancestral origin of an extant TSP shared by humans and one or more of the great apes was first documented about three decades ago when orthologous sequences from the highly polymorphic major histocompatibility complex (MHC) loci were compared between species [4, 6]. MHC (known as HLA, human leucocyte antigen, in humans) loci play a key role in the adaptive response to pathogens [7, 8], and some of the allelic lineages of the human HLA-DQ alpha locus (HLA-DQA1, HLA-DQA3 and HLA-DQA4) were deduced to have been present in the most recent common ancestor of the human, chimpanzee and gorilla lineages [4] and must therefore have survived for at least 8 million years [9, 10]. More recently, the human MHC lineage most strongly associated with delayed HIV-1 progression (HLA-B*57) was found to exhibit a high degree of similarity to a lineage frequently found among SIV-infected chimpanzees [11]. Other comparisons have reached a similar conclusion, namely that many alleles at the MHC locus have survived for an extended period of evolutionary time and hence are currently shared by multiple primate lineages. One example is provided by the MHC-DQB1*06 allele which predates the separation of the hominid and Old World monkey lineages more than 35 million years ago [12]. Shared alleles at the MHC locus have also been found in other primates such as the rhesus and cynomolgous macaques [13], between Madagascan lemurs in which some alleles appear to have been maintained for more than 40 million years [14] and between several non-primate lineages such as mice and rats [15], the brown bear (*Ursus arctos*) and the giant panda (*Ailuropoda melanoleuca*) [16], South American mouse opossums (*Gracilinanus microtarsus* and *Marmosops incanus*) [17] and between equines [18] among others (reviewed in [19]). The maintenance of these shared polymorphisms over such extended periods of evolutionary time implies strong selective pressure on the host immune response elicited by the pathogenic agent. However, it should be borne in mind that the potential contribution of recurrent mutation to the origin of these shared variants has not always been unequivocally excluded. It may therefore be that some of these ‘shared variants’ are actually identical-by-state rather than identical-by-descent. At the same time, direct evidence for a functional role for the balanced variant, or group of variants, is lacking in most cases. Although these *caveats* do not necessarily challenge the now well-established role of balancing selection acting on MHC loci, it precludes the acquisition of a clear picture of how often the TSPs are actually identical-by-descent as opposed to simply being identical-by-state.

Another well-established example of long-term balancing selection operating in the primate genome is provided by the ABO blood group locus. In humans, three main alleles account for the diversity at this locus, corresponding to the A, B and O blood groups. The A and B alleles are functionally distinguished by the co-occurrence of two missense mutations (Leu266Met and Gly268Ala, respectively) in the encoded glycosyltransferase whereas the human O allele results from an inactivating single-nucleotide deletion (261delG) that impairs enzymatic function, resulting in failure to convert H antigen into A or B [20]. A and B alleles are shared between humans and non-human primates [21–23]. Although one of the alleles has been lost in some lineages during great ape speciation (e.g. common chimpanzees and bonobos exhibit only the A antigen, whereas the gorilla harbours the B antigen), other lineages have retained both the A and B identical-by-descent alleles, e.g. the orangutan which shared a common ancestor with humans more than 16 million years ago [24]. Although pathogen-driven selective pressure operating on the balanced A/B alleles appears less intuitive than it perhaps is for the MHC locus, one must recall that there are several instances of histo-blood group antigens being associated with differential protection against multiple infectious microbes [25–27]. For example, infection by *Helicobacter pylori* has been shown to be reduced in A and B blood types as compared with carriers of the O type [25]. Moreover, a link between pathological conditions associated with *H. pylori* colonization, such as gastric [28, 29] and pancreatic cancer [30, 31], and the ABO phenotype has also been established in humans. Assuming that the persistence of the A/B polymorphism is maintained by pathogen-driven balancing selection, one may reasonably extend these considerations to the other great ape species.

### Beyond the MHC and ABO loci

Apart from the well-established examples of trans-species polymorphisms at the MHC and ABO loci which are maintained by balancing selection, few other properly substantiated examples of TSPs have been documented. One particularly interesting case is provided by the zinc-finger CCCH-type antiviral protein 1 (ZC3HAV1, also known as poly(ADP-ribose) polymerase 13-PARP13), a protein that is known to protect host cells from viral infection [32–35] and cellular stress [36]. The ZC3HAV1 polymorphic substitution Thr851Ile (rs3735007) is shared between humans and common chimpanzees and does not occur in a hypermutable CpG site (Table 1), supporting its candidacy as a true TSP [37]; an exhaustive analysis of the genomic region adjacent to this TSP has shown that the polymorphism has been selectively maintained in both species, probably as a result of its broad protective effect against viral infection.

**Table 1:** Properties and allelic frequencies of human single-nucleotide putative TSPs shared with bonobos and common chimpanzees

Gene	SNP ID	SNP flanking sequence^a^	MAF^a^	AA Change	Presence
*ZC3HAV1*	rs3735007	GTTTA(C/T)TGAAG	0.47 (C)	Thr851Ile	Human and common chimpanzee
*TRIM5*	rs34506684	TCTGG(C/T)GCCTC	0.46 (T)	Intronic	Human and common chimpanzee
*LAD1*	rs12088790	GGGCC(A/G)GCGAC	0.12 (G)	Leu257Pro	Human, bonobo and common chimpanzee

^a^Data extracted from dbSNP [69]

Another potential example of trans-species polymorphism under balancing selection is provided by the *TRIM5* gene. This gene also encodes an antiviral protein [38–40] (TRIM5, tripartite motif-containing 5), one that is known to act as a blocking factor of HIV-1 reverse transcription thereby limiting the efficiency of the infection in primates [41]. In non-primate species, TRIM5 is also active as an antiviral protein [42–45] and the reconstruction of TRIM5 evolutionary history has provided evidence for a long-term interaction with several different viruses prior to the origin of primate lentivirus [46].

A previous study has evidenced balanced TSPs in the primate *TRIM5* genes [47]. In a separate investigation, two intragenic *TRIM5* polymorphisms were found to be shared between human and common chimpanzee (Table 1): an intron 1-CTC insertion/deletion and an intron 1 transition (rs34506684), the latter occurring at an CpG dinucleotide [48]. Although the functional significance of these variants has not yet been established, the authors suggested that the intron 1 variant rs34506684 might impact transcription factor-binding sites leading to allelic differences in transcriptional activity which could underpin inter-individual differences in susceptibility to infection. However, because this shared polymorphism occurs at a hypermutable CpG site (Table 1), its candidacy as a true TSP must be in some doubt; in the absence of any convincing evidence that this variant is of direct functional significance, it may simply be a marker in linkage disequilibrium with another variant that is under balancing selection.

The discovery of these putative regulatory variants within intron 1 of the *TRIM5* gene between humans and the great apes provides evidence for the maintenance of balanced regulatory polymorphisms across species (as distinct from balanced coding sequence variants), as well as yet another example of a TSP which may impact the host pathogen response. Trans-species regulatory polymorphism has been functionally investigated at the MHC-DQA1 locus in eight non-human primates [49]. Numerous trans-species polymorphisms were identified within transcription factor binding sites in the MHC-DQA1 promoter region. Loisel et al. [49] assessed the functional consequences of these variants using a reporter gene assay and identified significant differences between baboon DQA1 promoter haplotypes in terms of their ability to drive transcription in vitro. Taken together with the high levels of sequence variation in this region, these findings suggest a role for balancing selection in the evolution of DQA1 transcriptional regulation in primates although the biological mechanism underlying the assumed increase in fitness remains unclear.

More recently, a scan of the human genome yielded good evidence for six non-coding genomic regions where ancestral polymorphisms shared between humans and chimpanzees have been under the influence of balancing selection [2]. The closest genes to these regions (*FREM3*, *MTRR*, *PROKR2*, *HUS1*, *IGFBP7* and *ST3GAL1)* are all to some extent related to the innate immune response, a finding which would concur with a balancing selection mode of evolution. For three of these six regions (near *HUS1*, *IGFBP7* and *ST3GAL1*), a regulatory role for the polymorphisms involved was demonstrated, indicating that physiological differences resulting from a balanced polymorphism can also be exerted at the level of gene expression. A role for regulatory variants as targets of balancing selection is not surprising since many studies have reported an important role for heterozygote advantage in the evolution of gene expression [50–52]. Although many of these variants may individually be associated with deleterious effects, they may nevertheless provide fitness advantages under certain environmental conditions [51, 53] by potentiating an optimal level of gene expression [52]. It is also quite possible that some of these regulatory balanced alleles have persisted for long periods of time in immunity-related genes thereby providing further likely examples of TSPs between humans and the great apes.

The most recently reported example of a putative TSP is that in exon 3 of the ladinin 1 (*LAD1*) gene (rs12088790) (Table 1) which has been claimed to be maintained by long-term selection in humans, common chimpanzees and bonobos [54]. However, once again, the shared polymorphism occurs at a CpG dinucleotide and hence may not in reality be identical-by-descent. The resulting missense change (Leu257Pro) influences the expression of *LAD1*: the minor allele, which occurs at a frequency of 0.12 in humans (Table 1), is associated with an increased level of *LAD1* expression. Irrespective of whether this is a direct effect, or whether the missense variant is in linkage disequilibrium with another polymorphic variant with a regulatory role, it provides further evidence for the important role of shared polymorphism in modulating gene expression.

The *LAD1* gene encodes a collagenous anchoring filament protein that serves to maintain dermal-epidermal cohesion and is associated with IgA bullous dermatosis, an autoimmune disease [55]. Apart from its pathogenic role in the context of IgA bullous dermatosis, there is no information as to how *LAD1* might contribute to the host response against a pathogen. However, many autoimmune diseases are triggered by infectious agents in addition to environmental factors [56], and this has been specifically reported to be the case in IgA bullous dermatosis [57].

### Overdominance vs. pathogen-driven frequency-dependent selection

The maintenance of TSP by long-term balancing selection has long been held to be mediated by heterozygote advantage (overdominance) or frequency-dependent selection [19]. Previous studies have claimed that the most important factor for the maintenance of MHC polymorphism is overdominant selection [58, 59]. In support of this postulate, MHC heterozygosity has been experimentally demonstrated to enhance resistance to multiple-strain infections [60]. However, a simulation-based approach has demonstrated that polymorphism at the MHC locus results not merely from overdominant selection but also from frequency-dependent host-pathogen coevolution for rare MHC alleles [61].

Host-pathogen interactions have also been proposed for the ABO TSPs whose maintenance over long periods of evolutionary time may have been due in part to coevolution with gut pathogens [62]. In accordance with this suggestion, a general model that integrates frequency-dependent selection and genetic drift would appear to account for the *ABO* polymorphism in humans [63]. Irrespective of the precise mechanism underlying the maintenance of a given TSP, it is important to appreciate that to be effective, the selective agent would need to exhibit the following properties: (a) it must be widespread geographically, (b) it must have been present over an extended period of evolutionary time, and (c) it must show similar tropism towards different yet evolutionarily related species. Further, heterozygosity at the targeted locus would have to mediate similar selective responses in the different species involved, and selection in favour of the heterozygote would have to be sufficiently intense to maintain the frequency of the minor allele(s) in the host species populations. The selective agent most likely to possess these properties is a pathogen. Alternative selective agents such as climate and diet would be most unlikely to remain constant over extended periods of evolutionary time [64].

## Conclusion

The presence of shared polymorphisms across evolutionarily related species is a strong indicator of balancing selection. Although most of the studies to date have focussed on the classical examples of the MHC and ABO loci, a few additional examples are known between humans and the great apes. Herein, we have reviewed these cases and noted that a common feature of virtually all well-established cases of balanced TSP is their association with the host-immune response, presumably triggered by infectious agents. The diversity observed at immunity-related loci is certainly shaped by the dynamic process of host-pathogen coevolution [65], and therefore, key aspects of a species’ adaptation to challenging environments are likely to have been pathogen-driven [66–68]. This raises the question of the potential relevance of TSP identification to improving our understanding of the host immune response; indeed, the study of these ancient variants could lead to new insights into immune system function with important implications for preventive medicine. Finally, it may be that the identification of additional balanced TSPs in humans and their closest relatives among the great apes might be facilitated by a guided search (e.g. by targeting immune system/autoimmune disease-associated genes specifically). The difficulty inherent in any such quest would be to prove that a newly detected shared polymorphism is identical-by-descent rather than simply identical-by-state. This would be especially true for polymorphisms residing within CpG sites where the balance of probability must lie firmly on the side of their being identical-by-state, at least until functional evidence to the contrary can be provided.

## Abbreviations

LAD1: ladinin 1
HLA: human leucocyte antigen
MHC: major histocompatibility complex
TRIM5: tripartite motif-containing 5
TSP: trans-species polymorphism
ZC3HAV1: zinc-finger CCCH-type antiviral protein 1
